# Dilemmas of care: Healthcare seeking behaviours and antibiotic use among women in rural communities in Nam Dinh Province, Vietnam

**DOI:** 10.1016/j.socscimed.2024.117483

**Published:** 2024-12

**Authors:** Yen Hong Thi Nguyen, Rogier van Doorn, Jennifer Ilo Van Nuil, Sonia Lewycka

**Affiliations:** a Oxford University Clinical Research Unit, Ho Chi Minh City, Viet Nam; b Oxford University Clinical Research Unit, Hanoi, Viet Nam; c Centre for Tropical Medicine and Global Health, Nuffield Department of Medicine, University of Oxford, Oxford, UK

**Keywords:** Vietnam, Antibiotic resistance, Healthcare seeking behaviours, Poverty, Inequality, Medical pluralism

## Abstract

Antimicrobial resistance is a silent pandemic to cause an estimated ten million deaths by 2050. Self-medication with antibiotics in low- and middle-income countries has been identified as a driver of antibiotic resistance. Interventions targeting solely individual behaviour change around antibiotic practices are often unsuccessful as they fail to address socio-cultural and structural causes of the problem. Understanding the context of antibiotic use in communities will better inform interventions addressing the misuse and overuse of antibiotics. Vietnam faces a growing threat of antimicrobial resistance due to inappropriate use of antibiotics in the healthcare system, farming and food production, and in the community. To understand the roots of this problem, we conducted qualitative research in 2020, with one component focusing on the community. This included fifteen in-depth interviews with women and four months of participant observation in three districts in Nam Dinh Province to explore the healthcare seeking practices and perceptions of medicine and antibiotic use. We argue that even when participants understood antibiotic resistance and were willing to adjust their care practices with antibiotics, there were cultural and structural challenges demotivating changes. The participants faced what we term “dilemmas of care”. For example, while public health messaging promoted appropriate antibiotic practices, the healthcare system did not provide a suitable environment to support appropriate use. Besides, the introduction of biomedicines into the long-standing traditional medical system caused confusions in community health practices, leading to issues such as poor adherence to treatment. At an individual level, participants faced challenges in accessing healthcare knowledge, adhering to social expectations surrounding care, and financial issues. We argue that the misuse of medicines and antibiotics in communities are responses to a deficient healthcare system and unequal access to quality healthcare.

## Highlights

• Women in rural communities use antibiotics to mitigate social relations and financial struggles.
• Medical pluralism may cause misuse of antibiotics in community.
• Communications on antibiotic resistance should be aware of local contexts.
• Antibiotics can be prescribed irrationally by medical doctors in primary healthcare.
• Unequal distribution of quality healthcare causes self-medication of antibiotics in communities.

## 1 Introduction

Antimicrobial resistance (AMR) is a silent pandemic associated with nearly five million deaths in 2019 (Murray et al., 2022), and that will reach an estimated ten million deaths by 2050 if there is no effective intervention (O'Neill, 2016). Inappropriate use of antibiotics in communities, for example for managing acute respiratory infections, has been reported widely as a driver of antibiotic resistance (Hoa et al., 2011; Larson, 2007; Mao et al., 2015). Examining self-care and self-medication, researchers found that patients, especially in low- and middle-income countries (LMICs), routinely self-medicated with antibiotics, had easy access to antibiotics over the counter, and often requested antibiotics from healthcare professionals (Do et al., 2021; Ocan et al., 2015; Okumura et al., 2002). Studies in Vietnam and other countries found that sociodemographic background, lack of health knowledge, misconceptions of antimicrobial resistance, and the influence of social relationships, like friends and family members, are the main influential factors for individual misuse of antibiotics in communities (Di et al., 2022; Fletcher-Miles and Gammon, 2020; Gautham et al., 2024). The process by which antibiotics were integrated into the traditional Vietnamese culture and used in the same way as traditional medicines, called “indigenisation” of antibiotics, has caused some local misunderstanding in the use of the medicines, such as reducing antibiotic doses (Halfvarsson et al., 2000).

Interventions to improve awareness of antibiotic resistance and improve practice of antibiotic use through mass media education have been implemented in high-income countries, but failed to provide clear evaluations of their impacts (Cross et al., 2017). Recent studies showed that interventions targeting individual behaviour change were often unsuccessful as they ignored structural factors influencing behaviours (Rodrigues, 2020; Willis and Chandler, 2019). Using a structural approach, Willis and Chandler (2019) argued that antibiotics have functioned as a “quick fix” for fragile infrastructure and inequality, and have been used to enable the productivity of people, farms and crops in LMICs (Willis and Chandler, 2019). Researchers have also criticized the term “inappropriate use” of antibiotics, which can be seen as pejorative and is often a claim that ignores structural aspects, and the specific circumstances and people's own reasoning or logic for using antibiotics. For example, Whyte et al. (2003) found that medicines have a “social efficacy” as they are not only expected to alleviate symptoms but also to restore the ability to carry out daily activities and work for patients (Whyte et al., 2003). Rodrigues (2020) examined self-medication and found that antibiotic consumption does not always follow biomedical recommendations, but instead depends very much on the socioeconomic and therapeutic landscape, in this case, in Mozambique (Rodrigues, 2020). Haenssgen et al. (2020) also found that labour precarity and social marginalization are determinants of people's misuse of antibiotics in rural Laos and Thailand (Haenssgen et al., 2020). Perceptions and practices of medicine misuse in everyday life in Tororo, Uganda might not just be defined by the local context, but also by the nation's political and economic visions of “tapping” wealth to encourage economic growth (Nayiga et al., 2022). In Vietnam, researchers have explored the practice of antibiotic use at individual levels, but there have been few explorations on how structural factors influence antibiotic use and resistance.

In this paper, by exploring healthcare seeking practice for acute upper respiratory infections, we attempt to look beyond individuals and identify socio-cultural and structural issues driving the use of antibiotics in communities within the Vietnamese rural context. We describe the daily care situation in the local communities as “dilemmas of care” to argue that even when the participants were aware of the dangers of antibiotic resistance and some of them were willing to adjust their current practice of antibiotic use, there were socio-cultural and structural challenges demotivating changes in their care practices. The use of medicines and antibiotics in the communities is a response to a vulnerable healthcare system and inequality. Our findings provide insights addressing broader socioeconomic and cultural issues for more sustainable behaviour change interventions.

### 1.1 Study context

Vietnam is a lower middle-income country in South-east Asia. Following a market-oriented economy launched in 1986, the healthcare sector started a new approach including changing from predominantly free access to healthcare to the introduction of paid healthcare services, legalisation of private pharmacies and clinical practices, and liberalisation of production and sales of pharmaceuticals. Legislation to ban over-the-counter (OTC) sales of prescription drugs was introduced in the Pharmacy Law of 2005 and further revised in 2016, but is poorly enforced, and in practice, OTC antibiotic sales without physician prescriptions are widespread. To provide better social protection to the people, the government introduced national social health insurance in 1992, which covered 93.00% of the population in 2023 (Pham, 2022). However, out-of-pocket expenses for healthcare remain high (Vuong et al., 2021).

The study was conducted in three Nam Dinh Province districts, south of the Red River Delta. The population is more than 1.8 million, of whom nearly 80% live in rural areas (Nam Dinh Statistics Department, 2023). According to the Nam Dinh Department of Statistics, the province had eight doctors per 10,000 inhabitants in 2022 (Nam Dinh Statistics Department, 2023). In 2022, the population in Hai Hau District was 276,005, in Xuan Truong District 163,875, and in Nghia Hung 184,645. At the time of data collection, Hai Hau, Xuan Truong and Nghia Hung Districts had one public hospital per district and one primary care centre in each of 72 communes. The public health system consists of primary healthcare at the commune level and hospitals at district, provincial, and national levels. Primary healthcare services include public health prevention, first aid, treatment of common ailments, diagnosing diseases, and making patient referral to higher healthcare levels. A referral system is required to transfer insured patients to higher-level hospitals when needed. However, since 2016, patients are able to use health insurance in hospitals at the district level in their province without a referral from the commune level according to the Revision of Health Insurance Law (Health Insurance Law No. 28/2008/QH12, Revision in 2020) (National Assembly of Vietnam, 2008). Drug counters, second-tier drug stores according to the Circular No. 02/2018/TT-BYT on Good Pharmacy Practice (Ministry of Health, 2018), either with or without Good Pharmacy Practice certificate (GPP) were easy to access from most villages. Data from local authorities showed that there were 159 pharmacies and drug counters in Hai Hau, 98 in Xuan Truong and 159 in Nghia Hung in 2022. Besides, each commune health centre also had a drug counter. In the study sites, there was a private healthcare system which consisted of private clinics, pharmacies, drug counters, private doctors and home-visit doctors (a local term was “bag-carrying doctors” because they carried bags to patient homes). In this paper, the private healthcare system refers to both licensed- and unlicensed healthcare facilities with registered- and unregistered healthcare practitioners.

### 1.2 Data collection and analysis

The study employed an ethnographic approach with a combination of participant observation and in-depth interviews. Our researcher (YHTN/Yen) spent four months in the study districts conducting in-depth interviews and participant observation from June 2020 to December 2020. Participant observation allowed her to experience first-hand healthcare seeking behaviours in the local community by participating in daily life, having informal discussions about healthcare and accompanying key participants to healthcare facilities. Fieldnotes were written in narrative style and analysed. As a female researcher, Yen was introduced to and invited to live with a woman in her seventies and her youngest daughter in her twenties. The host family and the neighbourhood became the main informants of the study.

We conducted one in-depth interview with a woman in each fifteen villages within the three districts. We chose to talk to women because women often take the main responsibility for taking care of their family's well-being, especially for young children (Craig, 2002; Gautham et al., 2024). Based on literature which identified that cough symptoms were often self-treated by community members (Dai et al., 2024; Lee et al., 2016), we assumed that upper respiratory infections do not require intensive negotiation to seek formal healthcare in our target communities. We used purposive sampling to prioritise women who had children and children under five years old, as they would have more experience with respiratory infections. To gain different perspectives, we tried to divide the sample into different age ranges, marital statuses, and occupations. We excluded participants under eighteen years old. Topics in the in-depth interviews included the participants' experiences in health-seeking for themselves, their family, and their children focusing on acute respiratory infections and their perceptions of antibiotic use and antibiotic resistance and healthcare in general. Before closing the interviews, we explained to the participants about antibiotic resistance and provided information regarding antibiotic use.

In the interviews, the researcher asked the participants to sort common medicines that we prepared in a drug bag. This activity was a visual method for us to explore if the participants could recognise antibiotics or not and what indicators they used to identify common medicines. To build the drug bag, the researcher visited three drug counters in Hai Thanh commune and asked the drug sellers to prescribe medicines for cold, flu, and some upper respiratory symptoms for adults and children (details of the medicine in Annex 1). The drug bag included four types of antibiotics (penicillin, amoxicillin, ampicillin and cefixime), painkillers (paracetamol, ibuprofen), anti-inflammatory drugs (alpha chymotrypsin), expectorants (acetylcysteine), antihistamines (loratadine), vitamin C, cough lozenges and a cough syrup. We tried to buy those drugs with a range of different brand names as the study participants might be familiar with certain packaging.

Before the exercise, the participants were given time to examine the medicines in the bag without any prior information. They then, sorted the medicines based on their understanding. During this sorting process, we discussed their knowledge of the medicines and the circumstances in which they had used them. After completing the activity, the researcher asked if they would like to redo the sorting in different ways. Most participants indicated that it was the only way they comprehended the medicines.

We relied on our local partners to recruit study participants. We consulted with the district study coordinators to choose study communes based on their proximity to the district hospitals and towns. To invite participants, we provided the head of the commune health centres with a list of criteria including age, number of children and children under five, and occupation for them to identify a suitable individual within the community. The interviews took place in a private space in the commune health centres or the participants’ homes. The researcher conducted the informed consent process before each interview. During the informed consent process, we provided the study information and gave the participants an opportunity to decide if they wanted to participate in the interview. We encountered only one case in which the participant was not well enough to participate in the interview, so we looked for a replacement in the commune. One time, a participant had to leave the interview early, and we returned to continue the interview another day. All participants gave written informed consent before the interviews. For participant observation, key participants were given study information and we audio-recorded their verbal informed consent.

All in-depth interviews were audio-recorded and transcribed verbatim for analysis. The researcher (YHTN) used Nvivo 12 – the software that helps to arrange data into patterns, categories, and themes – to analyse the transcripts and fieldnotes (Lumivero, 2024). We used thematic analysis to create codes and used inductive and deductive approaches simultaneously while creating the codebook (Braun and Clarke, 2006). Deductively, codes were derived from the interview guides and literature on social aspects of AMR (Saldaña, 2015). Inductively, codes emerged from the raw data (Saldaña, 2015). Similar codes were grouped into themes and formed relationships. The interpretation of the themes and relationships was done in the last stage with support from extensive reading on the topic. We also reviewed grey literature including regulations, laws, reports, news and communication materials on an ad-hoc basis along with the interviews and informal discussions to gain in-depth understanding of contextual factors in healthcare. All participant names mentioned in this manuscript are pseudonym.

The study was approved by the National Institute of Hygiene and Epidemiology (approval number NIHE IRB-29/2019) and the Oxford Tropical Research Ethics Committee (approval number 529-19).

### 1.3 Study participants’ characteristics

The ages of the fifteen participants ranged from 23 to 62 years old. As explained, we prioritised talking with women who had at least one child and had children under five. We, therefore, had a majority of participants in their twenties and thirties. In fifteen participants, two of them were single and thirteen of them were married. The participants' occupations included farmers, small business owners/staff, governmental staff, factory workers, school teachers and housewives. Details of participants’ characteristics are presented in Table 1.

**Table 1:** Study participants’ characteristics.

Participants' characteristics	Number of participants
*Age, years*	
20–29	6
30–39	4
40–49	3
50–59	1
>60	1
*Marital status*	
Married	13
Single	2
*Number of children/grandchildren*	
None	2
1	5
2	5
3	2
4	1
*Number of children under five*	
None	6
1	8
2	1
*Occupations*	
Farming	3
Business owner/worker	4
Factory worker	3
Part-time governmental staff	2
School teacher	1
Housewife	1
Retired	1

### 1.4 Theoretical approach

Our analysis was inspired by Whyte, van der Geest, and Hardon's “Social lives of medicines” suggesting that real life efficacies of medicines are “assessed not by pharmacologists, but by social actors, who have their own criteria and expectations” (Whyte et al., 2003, p. 23). More specifically, social efficacy explains the meaning of giving and taking medicines beyond their clinical purposes to those involved (Whyte et al., 2003). We employed Clifford Geertz's “thick description” to explore the meaning of antibiotics in everyday healthcare seeking practice (Geertz, 1973). “Thick description” is used to “characterize the intentional, communicative, interpretative meaning of the behaviour: why it was done, how it was read, and using which social codes.” (Luhrmann, 2015, p. 291). This interpretative approach allowed us to spin the mothers' practice of giving antibiotics into the webs of sociocultural, economic, and healthcare significance in order to make sense of the practice from local perspectives. We then, applied Annemarie Mol's “logic of care” to develop the “dilemma” argument (Mol, 2008). In her study about healthcare choices with diabetic patients, Mol (2008) suggests that patients fail to make ideal choices for care or treatment for two reasons: firstly, patients are often treated as objects and made passive, thus lack the ability to choose; and secondly, patients are often not provided sufficient material resources required to make ideal choices (Mol, 2008). Applying Mol's ideas, we recognise that mothers in the study, even when they wanted to choose not to give antibiotics to their children, did not have essential resources for making that choice. The concept of “dilemmas of care” is to capture the tension of making a choice to use antibiotics in the absence of conditions to choose otherwise.

## 2 Results

### 2.1 Healthcare seeking behaviours in rural communities

#### 2.1.1 Home practice and traditional remedies

Perception of illness severity was the basis for people's healthcare choices. When people had minor common symptoms such as cough, nasal congestion, or fatigue, some people would take a short rest and treat themselves with traditional and home remedies. Traditional remedies for easing coughs could be found in their gardens or easily bought from the local markets such as herbs, kumquats, lime, white rose petals, or honey. Herbal ointments were regularly used for multiple treatment purposes such as managing pain and itch. Traditional and home remedies were perceived to be natural and safe for treating minor ailments or relieving mild symptoms, but with slow effectiveness. If the symptoms persisted, people opted for solutions from Western biomedicines which were preferred for their strength and fast effectiveness.

#### 2.1.2 Self-medication and over-the-counter practice

**Fig. 1:**
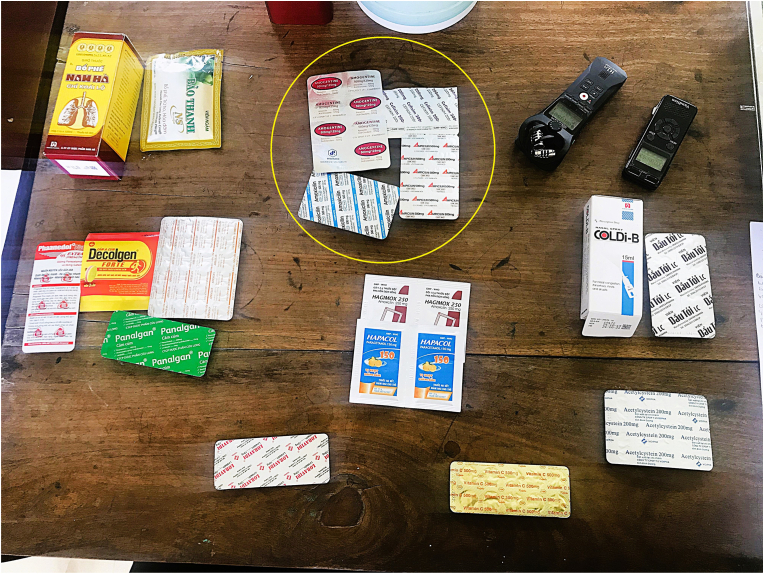
Medicines were grouped based on their mechanism of action. This participant separated antibiotics (in circle) from other medicines.

**Fig. 2:**
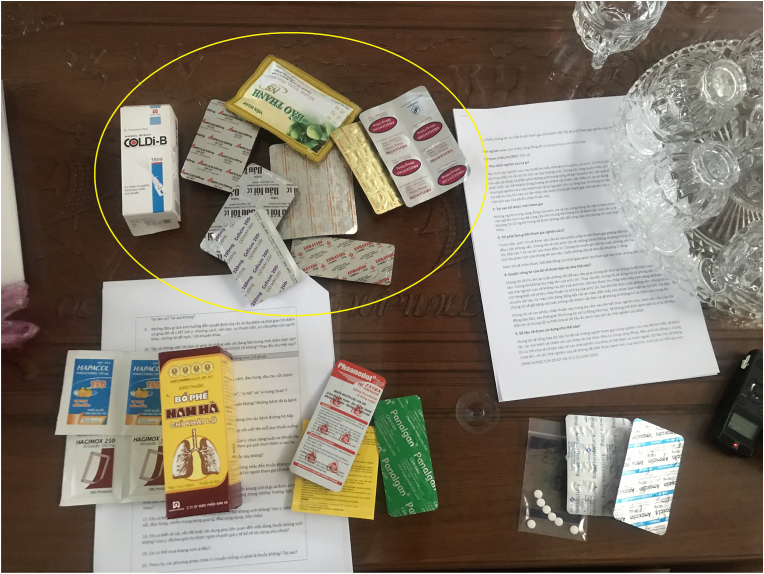
This participant could not recognise antibiotics and grouped them together with other medicines often prescribed for flu symptoms including antihistamine, vitamin C, cough lozenges (in circle).

Some participants sought biomedical treatment at the onset of symptoms, to reduce the duration of illness. People in the communities often self-medicated from their previous positive experiences or advice from relatives and friends. The medicine sorting activities demonstrated that participants showed varied understandings of antibiotics. In general, we found that younger women with children had better understanding of the provided medicines and antibiotics compared to others. Some participants could identify familiar antibiotics among other drugs and put antibiotics into a separate group (Fig. 1). Some participants arranged the medicines in combinations that they often received from pharmacies for respiratory infections (Fig. 2). Some recognised specific antibiotics by the names such as Amoxicillin or Cefixime as they had used them before. By not knowing antibiotics' active ingredients, most participants could only recognise antibiotics with familiar packaging or the sound of a drug's name. Antibiotics were often recognised by their red and green capsuled appearance. “Antibiotics often have an “i” in its name” – Mai, a mother of three, shared her technique to recognise antibiotics. While one participant demonstrated a good understanding of how antibiotics work, the majority were not able to articulate their function and uses. Perceptions of the medicines were closely linked to their perceived effectiveness in treating specific illnesses and symptoms, such as curing the flu, alleviating diarrhoea, combating infections, or relieving pain.

Biomedicines including antibiotics were ready to purchase over the counter. In most cases, people bought their medicines from drug counters or pharmacies by listing their symptoms to the store staff. Often, people did not finish the recommended course and saved the medicines for the next time.

#### 2.1.3 Seeking professional advice from home-visit doctors, private doctors and higher healthcare levels

While seeking treatment in pharmacies and drug counters was the most popular practice in the community, home-visit doctors or private doctors were the second option for most people when they needed medical support for early symptoms, especially for those who were too tired to go out or could not manage a health check during working hours. “Home-visit doctors”, who often had no university-level medical degree and gained medical knowledge through learning from authorised doctors, worked in the community without a formal license. Most participants could not clarify the home-visit doctors’ background. “Private doctors” were official healthcare staff who worked in the commune health centres or state hospitals and worked in private healthcare facilities or at home for additional income. The private doctors also visited patients at home when needed. They sold medicines directly to patients and gave injections to most patients. The popularity of this injection practice made the term “going for injections” an alternative for “seeing a doctor”, even though in many cases, people did not receive injections. Polyclinics, which were private healthcare facilities providing out-patient services and often equipped with a laboratory were popular in towns of the districts. In Hai Hau District, there were four polyclinics attracting patients within and from neighbouring districts. Then, if the symptoms persisted or got worse, most people would go to district hospitals or hospitals at provincial and national levels.

### 2.2 Dilemmas of care

An was a young mother of two boys. Being a typical mother in a rural commune in Hai Hau Province, An was the main caregiver in the family, and she was also working full time in her family carpentry workshop. To maximise profits, she did not hire many helpers, so she was often swamped with work. A drug counter which was just 200 metres away from her home was her “go-to” place when her children or other family members showed early symptoms of sickness. Her other options were a private doctor in the commune and a provincial or national hospital if the symptoms progressed. In the interview, An talked to the researcher about her healthcare seeking routine and they discussed antibiotic resistance – the term appeared unfamiliar to her at first. Although An knew quite well that antibiotics should be used for at least five days, she often cut the course short and saved them for the next time. She hardly recognised the common antibiotics in the sorting activity. Towards the end of the conversation, after explaining about antibiotics and the concept of antibiotic resistance, Yen – our researcher asked:

“Do you think you would change your habit of using antibiotics to manage your children’s respiratory infections?”

“No”, An answered sharply.

Yen made a short pause, then asked “why?”

“Because I don’t know what else to do. If I don’t give them antibiotics, my kids will not get well fast.” An answered.

(An, 32 years old, mother of two)

This short conversation between An and our researcher shows how having knowledge of antibiotic use and antibiotic resistance might not simply result in changing practice. Even though many mothers acknowledged that antibiotics are harmful to their children if used inappropriately and they were willing to adjust their use of antibiotics, in practice, they faced obstacles preventing change. Through exploring these apparent paradoxes and tensions, we developed the concept of *dilemmas of care* to illustrate the situation that happens when we situate the practice of antibiotic use in the broader context of socio-cultural, economic, and structural factors. We argue that the practice of antibiotic use in rural communities is socially, culturally, and structurally constructed. Therefore, what is seen as ‘inappropriate’ antibiotic use is a response to the healthcare structures and social norms people live in. By analysing “dilemmas of care”, we suggest shifting the lens to improving healthcare systems and reducing inequalities especially for poor and vulnerable groups, alongside promoting individual change.

#### 2.2.1 Dilemmas caused by public communication for antibiotic resistance and healthcare context

Public health messages about AMR created dilemmas. For example, messages such as “consult a healthcare worker before taking antibiotics” or “follow doctors’ instructions” was incompatible with the healthcare context of rural communities. To demonstrate this argument, we analysed examples used for public community communication for AMR and contextualised them within the context of antibiotic prescribing, and healthcare seeking behaviours in the rural areas in Vietnam.

**Fig. 3:**
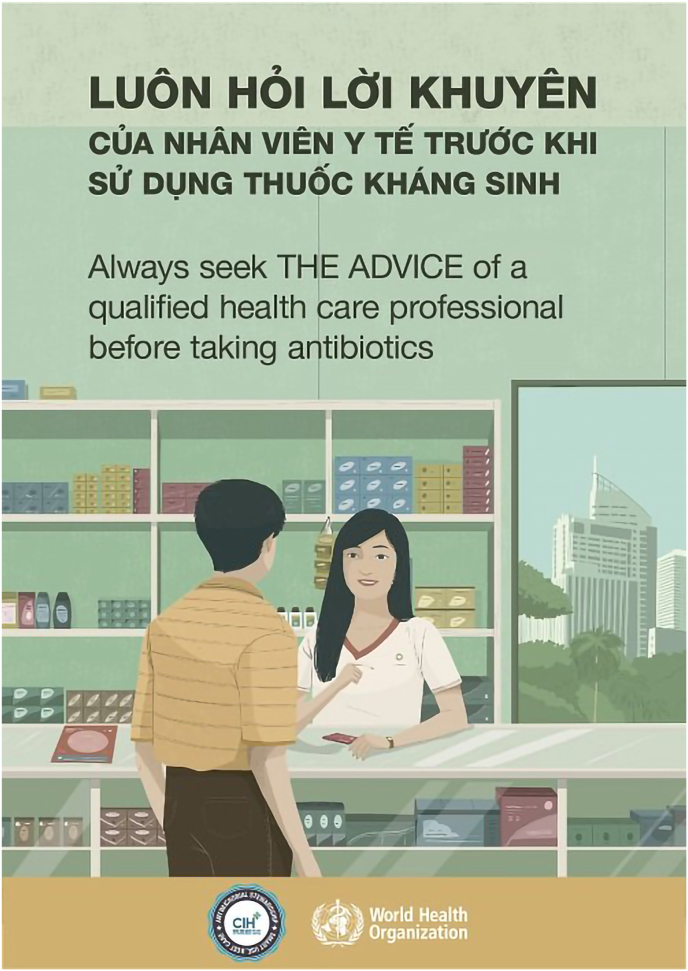
(City International Hospital, 2019): Examples of public health messages highlighting the action of seeking advice from healthcare workers before using antibiotics (WHO materials reused in Vietnam).

Public communication to raise awareness of antimicrobial resistance and to alter individual behaviours towards appropriate antibiotic use is promoted as an effective solution in the national strategy for prevention and control of drug resistance in Vietnam (Prime Minister of Vietnam, 2023). Communication strategies from the World Health Organisation (WHO) about antibiotic use and AMR targeted various areas and purposes including calling for collective action and responsibility to AMR; handling antibiotics with care by seeking advice from healthcare staff and incorporating a One Health theme (Rajkhowa and Thursky, 2020). In Vietnam, the public health messages from WHO were translated and reused in public communications (Fig. 3). “Seek advice from healthcare workers before taking antibiotics” seemed to be the most popular and well-received message; all study participants had heard the message before and were able to mention it in the interviews. In the rural communities where we did the fieldwork, posters or communication materials were hardly seen in public spaces or on television, but as people often looked for healthcare information on the internet, they might come across those messages without having a physical poster within their reach.

Although the message was successful in raising awareness that antibiotics should be prescribed by doctors, the definition of qualified healthcare professional was unclear in the communities. The term “đi bác sĩ” – “going to doctor” was widely used in various contexts, not knowing if it was doctors in official healthcare facilities who are authorised to prescribe antibiotics. In some interviews, it took time for the researcher to clarify the meaning of “doctors” they mentioned in their stories. In one case, “the doctor” was a neighbour who sold groceries and drugs at home. In many cases, the term “doctors” indicated staff in a pharmacy or a home-visit doctor who had no official antibiotic prescribing authorities. When the community members labelled whoever sells drugs and gives (even unlicensed) healthcare advice as “doctors”, they might understand the public health message differently from its original intended idea. Additionally, the broad term “healthcare workers” (nhân viên y tế) which is used in the above posters (Fig. 3) might create an idea that any healthcare worker could dispense antibiotics (City International Hospital, 2019). This idea is problematic in the context of rural Vietnam where antibiotics are often prescribed by healthcare staff who do not have enough knowledge for the action (Craig, 2002; Nguyen et al., 2019). The same mother in the above conversation – An – reacted defensively: “I did not decide to buy antibiotics by myself; I asked the drug counter's staff” indicating that she had already sought professional advice after the researcher advised her that antibiotics should be bought with a doctor's prescription. Fig. 3 illustrates a person talking with a healthcare staff standing behind a medicine counter which might also encourage the idea of buying antibiotics over the counter in the communities.

A deficient healthcare system in rural areas contributed to the dilemma that the people faced when making care-seeking decisions. While public health communication recommended people to seek professional advice before using antibiotics, in reality, access to professional advice was limited. For minor illnesses, community drug counters are more popular than primary care services. These are easily accessible from morning to evening any day of the week, but staff have limited qualifications to provide advice regarding use of certain medicines because they are only required to have two years of training in pharmacy. Furthermore, in practice, the sales staff in local pharmacies were often not qualified at all (Nguyen et al., 2019). A key informant revealed that she did not know if the drug seller in her most trusted drug counter, and probably the most trusted one for many in her village, had any pharmacological background. This drug seller had continued the business started by her husband who had some knowledge, but passed away a few years ago. So, this drug seller did not appear to be the healthcare professional that the posters referred to, but she was a typical case in the communities.

Primary healthcare is considered the most accessible level of the formal healthcare system, but restricted opening times for using health insurance still make visiting primary care centres less convenient than a drug counter. Even when people do visit primary care, they cannot avoid inappropriate antibiotics, as over-prescription of antibiotics is common. A large study of primary care records in Nam Dinh Province in 2019 found that 97% of patients presenting with acute respiratory infections were prescribed antibiotics in hospitals, commune health centres, polyclinics and pharmacies (Nguyen et al., 2020). Another social science study in Nam Dinh Province in 2019 showed that in 80% of the prescriptions, antibiotics were prescribed by healthcare staff in commune health centres for almost all diseases and conditions, regardless of whether or not it was appropriate (Hoang et al., 2019). The availability and quality of care in the healthcare system causes a dilemma, as even when people want to use antibiotics appropriately, the healthcare infrastructure does not support this. This reality makes the public health message - “seek the advice of qualified healthcare professionals” hard to apply and ineffective in reducing antibiotic consumption in this context.

#### 2.2.2 Dilemmas caused by socio-cultural, economic factors

A dilemma of care also happens when we juxtapose mothers’ love and their desire for the best care for their family with the social and economic conditions in which they live. Young mothers, in particular, believed that consuming too many medicines was bad for children who might suffer from side effects such as diarrhoea or upset stomach. To protect their children from the side effects of antibiotics or suffering from taking the pills, they were willing to learn new practices. However, mothers often found themselves in a conflicting situation where their willingness to use alternatives to antibiotics was prevented by external factors. In the following paragraphs, we demonstrate how contextual factors created dilemmas for mothers in their care responsibilities through analysing challenges related to access to biomedical healthcare knowledge and the socio-cultural and economic pressure. We also show why motherly love and desire to provide the best care for family members were situated in the dilemma of giving medicines and not giving medicines such as antibiotics.

##### 2.2.2.1 In the mist of medical pluralism

“Medical pluralism describes the availability of different approaches, treatments, and institutions that people use to maintain health or treat illness. Most commonly, medical pluralism entails the use of Western medicine (or ‘biomedicine’) and what is variously termed as ‘traditional medicine’ and ‘alternative medicine'.” (Khalikova, 2023, p. 1).

This section presents how understanding of biomedicine and traditional medicine caused misuse of antibiotics in the community through mixing up the practice nature of the different systems. Perceptions of bodily balance (yin and yang) in Chinese philosophy inherited in Vietnamese traditional medicines for generations were applied to biomedical practice. Although mothers thought that Western biomedicines were strong, fast, and effective, they also thought biomedicines would disturb the bodily balance and cause unwanted side effects. Therefore, they often sought biomedicines, including antibiotics, to relieve the symptoms quickly, but they shortened the recommended doses when there were no symptoms. Then they would switch to traditional or home remedies to maintain the yin-yang balance. While traditional and home remedies such as lime, honey, herbs were perceived as natural, biomedicine was referred to as “chemical”, “unnatural” and “toxic”. For that reason, motherly love was practiced through limiting antibiotic doses, but not necessarily appropriately.

Craig (2002) suggested the importance of the oral tradition in transmitting knowledge of household remedies within circles of family and friends in his study about Vietnamese medicines (Craig, 2002). The process relied entirely on memory. Those memories of traditional medical knowledge enable the experiences to travel through space and time. Nowadays, we found that the mothers in our study also kept memories of remedies and applied them in practice, even together with biomedicine. A mother shared that her “go-to” treatment for coughing was an antibiotic, a pain killer, and an anti-inflammatory drug which she could buy from a drug counter in her village.

“If there is no high fever, but there are a sore throat and coughing, if I cough a lot then I would have a headache, usually, I would take an antibiotic – Haginat (cefuroxime), and a Hapacol (paracetamol), along with an Alpha Choay (alpha chymotrypsin). I would take those for 5 days”. (Minh, 42 years old, mother of two)

Minh learned about the treatment after a visit to a doctor many years ago and it had become her knowledge for practice since then. She was confident when she talked about the practice because it worked most of the time. The mentioned medicines were personalised for Minh's family members, as Craig (2002) called it “familiar medicines” – a concept explaining the belief of “the right medicine for the right person” rooted from the practice of traditional medicines (Craig, 2002). The fact that mothers applied the practice of traditional medicine to biomedicine habituated the use of antibiotics in their care routine.

##### 2.2.2.2 Challenging access to healthcare knowledge

While traditional medicine and biomedicine knowledge systems conflicted with each other in practice, the challenging access to the Western healthcare knowledge including knowledge about antibiotic use added another layer of difficulty in giving care for study participants. Participants often chose to buy medicines in a trusted pharmacy or a drug counter which either looked nice or was run by someone in their acquaintance or had good reputation within their circle. Some mothers shared that they received consultation and sufficient advice on healthcare from staff in drug counters. However, actively asking for drug information, such as what they were and what they were for, was not a common practice by many mothers. They revealed that they did not ask for more details of the medicines given by the sellers because they would not understand and remember the information later. The mothers found this extra information was unnecessary as all they needed to remember was when and how the given medicines should be used. In some particular cases, we found trust and relationship played a role in this situation. In a few cases, asking back might indicate mistrust, so community people would not want to ask for more information to avoid the misunderstanding that they did not trust the healthcare worker. As the mothers went to familiar places, they trusted them with any suggestions without asking for more information as Mai shared below.

“Mai: Like, if I go to the pharmacy and ask them, they will answer, but I just don’t. They got used to it, so they would not … [give me further information]. I came there and we often chat about this and that, then someone else came so there was no time.

Yen: What did you chat about?

Mai: About our mutual acquaintance, then miscellaneous things.

Yen: Okay, miscellaneous things.” (Mai, 43 years old, mother of three)

Limited quality of lower-level healthcare might also limit access to healthcare knowledge. Healthcare services in rural or remote areas were often complained to have lower quality compared to those at higher levels (Quan and Taylor-Robinson, 2023). Some mothers felt confused when they received different advice for the same symptoms from doctors in provincial hospitals and in community primary care centres. To access higher level healthcare facilities, mothers faced logistical challenges such as long distance and transportation availability.

“From here to Thai Binh Province [Children’s Hospital] is about 12–13 km. There is not much difference in terms of distance [to the district hospital], but the provincial hospital is across the river from us, so we often must wait to take the ferry.” (Hoa, 46 years old, mother of one)

When mothers did overcome the inconvenient transport and the long distance to take their children to national or provincial hospitals they had an expectation for better consultation and healthcare advice. However, Vietnamese culture of hierarchical relationships between doctors and patients has been an obstacle for shy patients to actively approach doctors ( A. L. Tran et al., 2020). The mothers revealed that they did not actively request information about the diseases or the medicines they received because they did not want to disturb busy doctors. When patients did not ask, they did not receive more healthcare information from the doctors. Mothers’ narratives indicated that doctors did not show initiative to give patients healthcare information. Some participants complained about doctors in hospitals not paying attention and not providing information regarding their health.

“That’s right, if they [doctors] care about you, they would tell you more about your health and you would remember that.” (Thuy, 37 years old, mother of two)

In addition to formal healthcare providers, mothers also faced the challenge of having limited access to trusted healthcare resources online, for example, while using their phones. While the internet conveniently offered various sources of healthcare information, mothers found it difficult to tell which information was reliable. In the context of inadequate access to healthcare information leading to lack of healthcare knowledge, mothers relied on their personal experiences more than official health information and often settled with OTC medicines including antibiotics. Therefore, the medicines became a “quick fix” for good care and good health (Willis and Chandler, 2019).

##### 2.2.2.3 Socio-cultural pressure of being a good mother

Having inadequate healthcare knowledge caused mothers to feel anxious when their children were sick, especially when their children were young. Most mothers' greatest concern was that their children's condition would deteriorate if they did not use antibiotics from the early symptoms.

“What if my sickness or my child’s condition gets worse, so I cannot change [practice of antibiotic use].” (Thuy, 35 years old, mother of two)

Mothers found that giving antibiotics or medicines was a remedy for their anxiousness and uncertainty when their child was ill.

“Honestly, I become anxious when my children are seriously ill, and I see that antibiotics help them recover quickly” (Nga, 28 years old, mother of two)

Additionally, mothers were under pressure from other family members, such as their parents, or neighbours on how care should be given.

“There was a time when I hadn’t given him any medicine yet, and his grandparents wondered why his illness was lasting for so long. If I had already given him some medicine, they would wonder why he hadn’t recovered and kept on coughing after taking the medicine for so long. They would urge me to give him some other medicine. That’s something they say frequently.” (Thuong, 30 years old, mother of two)

In this mother's case, medicines were not only a possible treatment for her child but also saved her from the invisible pressure caused by her parents-in-law who also believed that medicines were the solution for their beloved grandchild. Indirectly, mothers providing medicines to the child also relieved the grandparents' concern about their grandchild's health. Medicines or particularly, antibiotics, carried a social meaning apart from their scientific one by mitigating family dynamics, especially in inter-generational households like Thuong's. And for mothers, giving medicines to an ill child was socially perceived as “appropriate” and being a “good mother”. Providing medicines to ill children symbolized good care and motherly love in this context.

##### 2.2.2.4 Economic pressure of poverty

Poverty and economic concerns were other factors influencing healthcare seeking behaviour and mothers’ decisions on giving antibiotics. Thoa who was a 55-year-old grandmother said that she was afraid to shower in the winter because her house neither had a room heater nor a water heater. She would be shaking hard after showering, so she would stay in bed to warm herself up. She shared that her body was weak and got sick easily. Therefore, she stocked a dozen Ampicillin packs at home to treat herself for mild ailments.

Thoa: This is an antibiotic.

Yen: Ampicillin. How do you know it’s an antibiotic?

Thoa: I took it many times.

Yen: You see the red pills?

Thoa: I have taken this medicine a lot my whole life. There are always dozens of these blister packs in my house.

Yen: Dozens of ampicillin packs?

Thoa: I take this medicine when I have a sore throat. When I am mildly ill, I take this medicine.

Yen: So, you take this medicine whenever you have a mild sore throat?

Thoa: When I have a sore throat or when I have a painful swelling.

(Thoa, 55 years old, grandmother of four)

The conversation demonstrates Thoa's frequent self-medication with antibiotics, which she perceived to compensate for her poor living condition and resulting sicknesses. A dozen ampicillin packs in her house offer assurance and a cheaper solution than a room heater.

The financial challenge not only appeared through using antibiotics as an alternative for poor living conditions, but also a cause for shortening the recommended doses. From our observations in a village drug counter, we saw that the drug seller asked customers their preferred number of doses, as it depended on their financial capacity. Mothers in the interviews talked about “tiếc tiền” (afraid of wasting money) to indicate people who cannot afford recommended doses. The phrase sometimes appeared negative with a judging expression to those who regard money above health. “Tiếc tiền” was also the reason for avoiding going to doctors or health check-ups, especially in adult patients in the community. Travel costs, examination costs, and medicine costs in hospitals were weighed with the severity of symptoms.

“My parents often urged me to take my child to Thai Binh Hospital when he was sick. But sometimes, his symptoms were mild, and the sickness was simple, we did not have to make a fuss. The doctor would return us home anyways. Going to hospital takes time, effort, and money; it’s not that all is free. Doctors will request an x-ray, then lab tests; so in most cases it’s a waste of money. It’s doing no good.” (Xuan, 38 years old, mother of one)

“Tiếc tiền” does not apply to Xuan's case as she tried to make a reasonable justification of her son's health condition before seeking professional help. But her story also shows that seeking quality healthcare is often not an easy decision to make, considering the time, effort, money, and pressure from others involved. Even in some severe cases requiring hospitalisation, mothers still had to balance their time, resources, and caregiving.

“[My child] took so many drugs, so much that he was afraid of drugs, that me as a mother, felt pain looking at it. Then, I told myself, okay, the next day, we would go to Nam Dinh [provincial hospital]. When we got to Nam Dinh, the doctors examined him, sold us drugs and as his symptoms were severe, they asked if I wanted to hospitalise my child, then I told them that I was very busy, I didn’t have the time and resources for that, so they sold me drugs to treat my child at home. The ones who sold me the drugs also told me to take for two days … three days, if my child wouldn’t get better then go to the hospital immediately. But then my child got better, so we didn’t go anywhere else.” (Mai, 43 years old, mother of three)

Additionally, Mai, like some other mothers, was afraid to lose her productivity if her child did not recover fast and needed care.

“I mean, it is … but … You gotta understand, it’s the rural area, I got work to go to. If my children are sick, they fuss a lot, they cry, it’s really annoying. I would just want them to recover fast so we can go to work.” (Mai, 43 years old, mother of three)

In the context in which antibiotics are considered magic bullets giving fast recovery, fast recovery means returning to work rapidly and earning money for living. The mothers’ love to give good healthcare by practicing appropriate use of antibiotics was pushed behind the financial priorities of the family. Antibiotics become a strategy to balance their motherly desire to be able to provide for their family financially with the desire for better quality healthcare.

## 3 Discussion

The study explored the healthcare seeking practice and perceptions of care and antibiotics of female community members in Nam Dinh Province. We found that with love and the desire to give the best care for the family, the mothers in the study were willing to adjust their practice of antibiotic use for their children, but there were challenges that made them hesitate to change. Those challenges included confusing information provided by public health communications on antibiotic resistance, their unique understanding of healthcare practice influenced by medical pluralism, the social pressure of being a good mother, and the difficult balance of financial care and health care that the mothers had to cope with. We termed these situations “dilemmas of care”.

While the arguments for blaming the individual for self-medication of antibiotics and the dangers linked to antimicrobial resistance are common (Sachdev et al., 2022), we argue that individual behaviours towards antibiotic use are structurally constrained by poverty and inequality. In Vietnam, Nguyen and Tomson found that self-medication has become a norm since the “Doi Moi” policy deregulating and privatising healthcare was implemented in 1986 (Nguyen and Tomson, 1999). The policy enabled the availability of medicines and increased access to medicines for the population through a dense network of private pharmacies (Beardsley et al., 2023; Nga et al., 2014). However, due to the lack of enforcement of prescription medicine regulations and the benefit-oriented healthcare business, antibiotics were easily bought over the counter and over-prescribed in healthcare facilities (Nga et al., 2014; Nguyen and Tomson, 1999).

The Doi Moi movement also resulted in reduced government funding for healthcare in general and a declining quality of primary healthcare, while famous hospitals in the cities were able to gain profit due to a large patient pool coming from rural and urban areas (Bloom, 1998). This resulted in an expanding gap of unequal access to quality healthcare between these areas (Bloom, 1998). A recent study on antibiotic prescribing practice in Nam Dinh Province indicated that primary healthcare failed to attract patients due to regular disruptions of medicine supply, low-quality doctors, limited funding as well as an insufficient health insurance payment cap (Vu et al., 2024). While patients hesitated to opt for poorly-equipped commune health centres, the availability of “high-tech” customer-oriented private clinics in both urban and rural areas has become their preference, even though service fees were applied (Lakin et al., 2024).

As people hesitate to buy health insurance because of poor quality primary healthcare, they might face financial trouble from bypassing lower-level healthcare facilities to access better healthcare in a higher-level facility. This makes social health insurance, which works on referral mechanisms, become useless for people living in vulnerable situations whose healthcare seeking involves constant economic calculations over quality of care (Dao, 2020, 2023; Nguyen Le Thao et al., 2022). Vuong et al. (2021) found that unequal geographical distribution of hospitals and healthcare quality in big cities and other regions discouraged people living in rural areas from using their health insurance in their locale and encouraged out-of-pocket payment at “higher quality” facilities further away (Vuong et al., 2021). This broader context of healthcare in Vietnam, with widely available OTC medicines, poor drug market management, and challenges in using social health insurance to access formal healthcare, supports our findings that self-medication with medicine bought from a drug counter is perceived to be the most economic, effective, and convenient approach to healthcare for people living in rural contexts. OTC purchase of antibiotics as an alternative to complex and expensive formal healthcare has been reported in other studies in Vietnam and in different resource-limited settings (Adhikari et al., 2021; Chowdhury et al., 2019; McKinn et al., 2021). The Vietnamese government's plan to develop primary healthcare in the future might strategically improve access to quality healthcare and reduce misuse of antibiotics in communities (Government News, 2024).

Many social science studies that use a contextual approach to antibiotic consumption find that medicine has social meanings and is affected by social relations (Gautham et al., 2024; Nayiga et al., 2022; Whyte et al., 2003). Antibiotics as a “quick fix” – the concept developed by Willis and Chandler (2019) to explain that antibiotics were used as infrastructure to deal with care in fragile health systems, with productivity in human and farming, with hygiene in limited-resource environments and with inequality in the context of political and economic violence - was mentioned several times in the findings as we found the resonance of the concept to our analysis in which antibiotics became “quick fixes” for a mother's social pressure of care, challenging access to a quality healthcare services, and balancing health care and financial care for the family (Willis and Chandler, 2019). While the notion of care is often associated with women (Craig, 2002; Gautham et al., 2024), we found that caregiving with antibiotics has helped women to navigate their way in patriarchal and hierarchical families. In our study, medicines and antibiotics could regulate relationships between mothers and their parents (in-laws), either easing or intensifying, by giving or not giving medication to an ill child. This indicates a holistic intervention approach in healthcare that must go beyond individuals and consider cultural influences and social structures (Aubel and Chibanda, 2022). Additionally, gender perspectives indicated that women, especially in low-resource settings, may be at a higher risk of contracting drug-resistant infections compared to men, primarily due to their menstrual hygiene needs and the division of labour (Wong, 2024). Despite this, the design of AMR interventions often neglects to address these gender disparities (Wong, 2024).

Rodrigues (2020) urged to shift the critique of “inappropriate” self-medication of antibiotics to pay attention to the socioeconomic and therapeutic landscapes of Maputo that shaped the practice (Rodrigues, 2020). Similar to our findings, the author pointed out that the sociocultural distancing between medical doctors and patients impacted communication regarding antibiotic knowledge and that the therapeutic practice of antibiotic over-prescription and prescription errors by doctors in formal healthcare facilities questioned the definitions of “rational” and “legitimacy” (Rodrigues, 2020). Shedding light on economic and political aspects, Nagiya et al. (2022) demonstrated that people's use of medicines, and antibiotics in particular, was their strategy to mitigate risks of everyday life and enhance opportunities for themselves towards wealth which was a contemporary ideology of the Ugandan government (Nayiga et al., 2022). Antibiotics, therefore, filled the gap of structural weaknesses and enabled community members to “tap” into national and global missions of being wealthy (Nayiga et al., 2022).

Medical pluralism has been an important topic in healthcare research (Khalikova, 2023). The concept of “indigenisation” of Western medicines to describe the way biomedicines are used after being incorporated into a local culture has been widely discussed (Haak and Hardon, 1988; Halfvarsson et al., 2000; Monnais and Tousignant, 2006). In Vietnam, Harfvarsson et al. (2000) focused on the use of antibiotics in everyday life and found that antibiotics were practiced in the same way that people used traditional medicines, resulting in shortened duration of antibiotic use (Halfvarsson et al., 2000). Several studies in Vietnam also found that the perceptions of “hot and cold” and “yin and yang” caused problems in drug adherence as biomedicines were thought to be strong and chemical and to cause bodily imbalance (Craig, 2002; A. L. T. Q. Tran et al., 2020). The way people used antibiotics to alleviate symptoms instead of prioritising diagnosis (McKinn et al., 2021) was also influenced by the traditional treatment principle aiming to relieve symptoms (Halfvarsson et al., 2000).

Understanding the local culture and situation might help to improve the effectiveness of public communication targeting antibiotic misuse, as messages might give rise to unintended consequences or prove ineffective in specific contexts. Huttner et al. (2019) examined the elements of antibiotic awareness campaigns on national and global levels in 2016 and gave an example that the message “Do not save left-over antibiotics” to target the issue of antibiotic self-medication would be inappropriate in the context of limited access to medicines (Huttner et al., 2019). Additionally, the confusing language used in public communication has also been critiqued (Mendelson et al., 2017). For example, a survey by WHO in 2012 found that the public was more familiar with “antibiotic resistance” or “drug resistance” than “antimicrobial resistance” (Mendelson et al., 2017). We also faced similar confusion in using specific terms for antibiotic resistance while communicating with the participants. “Lờn thuốc” or “nhờn thuốc” – local indications for a body that is less responsive to a certain drug - were more understandable for the community than the official term “kháng thuốc kháng sinh” (antibiotic resistance) which has always been used in public communication materials. This questioned if the local words or the official term should be used in communication campaigns, as the former would enhance the local relevance, but the latter would be more helpful in conveying the correct mechanism of antibiotic resistance (i.e. the bacteria, not the body becoming resistant to the drug), and for the population to gain familiarity with national-level or global-level campaigns.

## 4 Limitations

The concept of “dilemmas of care” is based on experiences of mothers in rural areas; for this reason, the argument is not entirely translatable to other community members. We acknowledge a limitation of missing male perspectives, as cultural influences of care would differ between men and women. However, based on the wider literature, we believe that men in the community also face some structural challenges to care and antibiotic use identified in this study.

## 5 Conclusion

The study demonstrates the limited choices that Vietnamese mothers in rural communities had when they practiced care and sought out healthcare. By analysing the socio-cultural context of care and healthcare seeking, we argued that these mothers faced different dilemmas when they wanted to adjust their practice of antibiotic use, including: unravelling correct ways to use antibiotics from the confusing public health message “see a doctor before using antibiotics” in the context of many “doctors” many of whom prescribe antibiotics unnecessarily; mixing up the principles of biomedical and traditional medicine; difficulties accessing healthcare knowledge; managing social pressure from others; and balancing the desire to give the both the best health care and the best financial to their children and families. We found that the misuse of antibiotics in the community was not simply an individual issue, but occurred within layers of other larger issues including cultural influences of care, insufficient drug management, unequal distribution of quality healthcare, and lack of an effective safety net and poverty. In conclusion, we recommend applying Mol et al.‘s “ethics of care” (2010), which amplifies the importance of seeking local solutions to specific problems rather than relying solely on generalised principles (Mol et al., 2010). Interventions aimed at reducing antibiotic misuse in communities should address these dilemmas of care and adopt a contextual approach that considers the diverse needs of stakeholders and addresses underlying structural challenges.

## CRediT authorship contribution statement

**Yen Hong Thi Nguyen:** Writing – review & editing, Writing – original draft, Methodology, Investigation, Formal analysis, Data curation, Conceptualization. **Rogier van Doorn:** Writing – review & editing, Supervision. **Jennifer Ilo Van Nuil:** Writing – review & editing, Supervision, Methodology, Conceptualization. **Sonia Lewycka:** Writing – review & editing, Supervision, Methodology, Funding acquisition, Conceptualization.

## Ethics approval statement

The study was approved by the National Institute of Hygiene and Epidemiology (approval number NIHE IRB-29/2019) and the Oxford Tropical Research Ethics Committee (approval number 529-19). All participants gave written informed consent before the interviews. For participation observation, key participants were given study information and we audio recorded their verbal informed consent.

## Figures and Tables

**Table:** 

Photos of drugs used in sorting activity	Names of drug	Active ingredients	Manufacturers
	Hagimox 250	Amoxicillin 250 mg (antibiotic)	DHG Pharmaceutical
	Cefixim 200 mg	Cefixim 200 mg (antibiotic)	COPHAVINA Pharmaceutical
	Ampicillin 500 mg	Ampicillin 500 mg (antibiotic)	THEPHACO Pharmaceutical
	Amogentine	Amoxicillin 500 mg Acid Clavulanic 125 mg (antibiotic)	PHARBACO Central Pharmaceutical
	Acetylcystein 200 mg	Acetylcysteine 200 mg (expectorants)	VIDIPHA Pharmaceutical
	Alpha chymotrypsin éloge	Alphachymotrypsin 4200 (anti-inflammation)	Eloge France Vietnam Pharma
	Decolgen Forte	Paracetamol (Acetaminophen) 500 mg (painkiller)	United International Pharma
	Phaanedol Plus Extra	Paracetamol 500 mg Caffeine 65 mg (painkiller)	USA-NIC Pharmaceutical
	Panalgan	Paracetamol 500 mg (painkiller)	Cuu Long Pharmaceutical
	Hapacol 150	Paracetamol 150 mg (painkiller)	Hau Giang Pharmaceutical
	Aphaxan	Ibuprofen 200 mg Paracetamol 325 mg (painkiller)	Armephaco Pharmaceutical
	Loravidi	Loratadine 10 mg (Antihistamines)	VIDIPHA Pharmaceutical
	Vitamin C	Vitamin C 500 mg	VIDIPHA Pharmaceutical
	COLDI-B	Oxymetazoline 7.,5 mg Menthol natural 1,5 mg	Nam Ha Pharmaceutical
	Bảo Thanh	Traditional herbs (Cough lozenge)	Bao Linh Pharmaceutical
	Bổ phế Nam Hà Chỉ Khái Lộ	Traditional herbs (Cough syrup)	Nam Ha Pharmaceutical
	Dầu tỏi	Garlic oil	Santex Pharma

## Data Availability

We will consider sharing our codebook or anonymised data upon request.
